# Application of machine learning to predict the occurrence of arrhythmia after acute myocardial infarction

**DOI:** 10.1186/s12911-021-01667-8

**Published:** 2021-11-02

**Authors:** Suhuai Wang, Jingjie Li, Lin Sun, Jianing Cai, Shihui Wang, Linwen Zeng, Shaoqing Sun

**Affiliations:** Department of Cardiology, The First Affiliated Hospital of Harbin Medical University, 122 Postal Street, Nangang District, Harbin City, Heilongjiang Province China

**Keywords:** Machine learning, Arrhythmia, Acute myocardial infarction

## Abstract

**Background:**

Early identification of the occurrence of arrhythmia in patients with acute myocardial infarction plays an essential role in clinical decision-making. The present study attempted to use machine learning (ML) methods to build predictive models of arrhythmia after acute myocardial infarction (AMI).

**Methods:**

A total of 2084 patients with acute myocardial infarction were enrolled in this study. (All data is available on Github: https://github.com/wangsuhuai/AMI-database1.git). The primary outcome is whether tachyarrhythmia occurred during admission containing atrial arrhythmia, ventricular arrhythmia, and supraventricular tachycardia. All data is randomly divided into a training set (80%) and an internal testing set (20%). Apply three machine learning algorithms: decision tree, random forest (RF), and artificial neural network (ANN) to learn the training set to build a model, then use the testing set to evaluate the prediction performance, and compare it with the model built by the Global Registry of Acute Coronary Events (GRACE) risk variable set.

**Results:**

Three ML models predict the occurrence of tachyarrhythmias after AMI. After variable selection, the artificial neural network (ANN) model has reached the highest accuracy rate, which is better than the model constructed using the Grace variable set. After applying SHapley Additive exPlanations (SHAP) to make the model interpretable, the most important features are abnormal wall motion, lesion location, bundle branch block, age, and heart rate. Among them, RBBB (odds ratio [OR]: 4.21; 95% confidence interval [CI]: 2.42–7.02), ≥ 2 ventricular walls motion abnormal (OR: 3.26; 95% CI: 2.01–4.36) and right coronary artery occlusion (OR: 3.00; 95% CI: 1.98–4.56) are significant factors related to arrhythmia after AMI.

**Conclusions:**

We used advanced machine learning methods to build prediction models for tachyarrhythmia after AMI for the first time (especially the ANN model that has the best performance). The current study can supplement the current AMI risk score, provide a reliable evaluation method for the clinic, and broaden the new horizons of ML and clinical research.

*Trial registration* Clinical Trial Registry No.: ChiCTR2100041960.

**Supplementary Information:**

The online version contains supplementary material available at 10.1186/s12911-021-01667-8.

## Introduction

Admittedly, AMI is a clinically critical disease [1]. Recent studies have emphasized that percutaneous coronary intervention (PCI) can reduce acute and long-term mortality [2]. However, the 1-year mortality rate for AMI patients reported by the Angiography Registry is still 10% [3]. Arrhythmia accompanying AMI is an important cause of worsening heart function and increased mortality [4–6]. Studies have confirmed that in patients undergoing PCI treatment, arrhythmia that occurred before and after the end of cardiac catheterization was associated with increased mortality [7]. As a result, identifying the risk factors of arrhythmia after AMI and predicting the occurrence of arrhythmia in AMI patients can arouse doctors' alertness and improve the prognosis of patients. In recent years, many studies have been concentrated on the risk factors of arrhythmia after AMI, including the clinical characteristics, coronary angiography results, and laboratory indicators [7–11]. However, the above studies are limited to a small number of factors and lack a comprehensive and multi-dimensional systematic evaluation of patients with arrhythmia in the acute phase of AMI. The GRACE risk score [1] is the most commonly used systematic assessment method for AMI patients, while it is mainly used to predict mortality, and the accuracy of predicting arrhythmia may not remain high. Therefore, establishing a predictive model of arrhythmia after AMI exerts an essential role in assisting clinicians in decision-making. Traditional risk models are usually based on statistical methods, which can only linearly analyze several factors' relationships. Researchers will select variables in advance to artificially cause the loss of potential risk factors. In terms of complex diseases such as acute myocardial infarction, it has higher requirements for dealing with multi-factor and multi-level interactions.

As the most critical subset of artificial intelligence, ML has gradually become an important research method in medicine [12–14]. Through simulating human learning activities, ML automatically obtains information from big clinical data for learning [15, 16], effectively avoiding the limitations of human factors and variables in traditional analysis. ML has been successfully applied in various cardiovascular field aspects, including disease prediction [17–21] and diagnostic classification [22–24]. In recent years, research on ML in AMI has mainly focused on predicting patient mortality [25–28]. In the field of arrhythmia, ML is mainly used for classification [29, 30], but the related ML model of arrhythmia after AMI has not been explored. As a result, this study intends to apply machine learning algorithms, including decision tree, RF, and ANN to establish a model to predict tachyarrhythmia after AMI and compare the performance with the model-based by GRACE risk variable set.

## Methods

### Patient cohort

We retrospectively studied patients with acute myocardial infarction diagnosed in the cardiac care unit of the First Affiliated Hospital of Harbin Medical University from January 2014 to January 2019. The guidelines define acute myocardial infarction as elevated Troponin I (TNI) (≥ 0.03 μg/L) or elevated Troponin I (TNT) (≥ 42 ng/L), accompanied by one of the following conditions: (1) Symptoms of myocardial ischemia; (2) New ischemic ECG changes: (3) Development of pathological Q waves; (4) Imaging evidence of new loss of viable myocardium or new regional wall motion abnormality in a pattern consistent with an ischemic etiology; (5) Identification of a coronary thrombus by angiography.

All patients underwent three-dimensional echocardiography, coronary angiography, and 24-h Holter. Outcome events were defined as whether or not tachyarrhythmia occurred. Arrhythmic events include atrial arrhythmia (atrial fibrillation, atrial flutter, and frequent atrial premature), ventricular arrhythmia (ventricular tachycardia, ventricular flutter, ventricular fibrillation, and frequent premature ventricular), supraventricular tachycardia. (All data is available on Github: https://github.com/wangsuhuai/AMI-database1.git).

### Variable selection

We selected the risk factors for tachyarrhythmia after AMI identified in the previous study, and added some new risk factors as candidate variables, including demographics, admission baseline characteristics, laboratory characteristics, echocardiographic parameters, and angiography Features, a total of 45 variables (Table 1), all variables were collected immediately after hospitalization and before PCI. As some patients received emergency PCI, the 24-h Holter record includes data before and after PCI. We graded continuous variables and converted them into ordered categorical variables (see Additional file 1).

**Table 1:** Variables for machine learning

Category	Variables
Demographics and medical history	Age, sex, smoker, drinker, Pre-hypertension, Pre-diabetes Mellitus, Prior MI, Prior CI, Prior HF, Prior CHD
Baseline characteristics of admission	SBP, DBP, HR, Killip, NYHA
Laboratory characteristics	Pro-BNP, CRP, Total cholesterol, Triglyceride, HDL, LDL, Cr, K + , TNI, CK-MB, UGLU, DDP
Findings on ECG	P-R, QTc, BBB (LAFB, LPFB, LBBB, RBBB)
Echocardiographic parameters	LVEF, FS, E/A, Dt, LVEDD, IVST, LVPWT, LA, RA (up and down), RA (right and left), PA, Vpa, Vao, ventricular wall motion
Angiographic characteristics	LAD, LCX, RCA, LM, LAD + LCX, LAD + RCA, RCA + LCX, Triple vessels

MI indicates myocardial infarction; CI, cerebral infarction; HF, heart failure; CHD, coronary heart disease; SBP, systolic blood pressure; DBP, diastolic blood pressure; HR, heart rate; pro-BNP, pro-B-type natriuretic peptide; CRP, C-reactive protein; HDL-C, high-density lipoprotein cholesterol; LDL-C, low-density lipoprotein cholesterol; DDP, D dimer; Cr, creatinine; TNI, Troponin I; CK-MB, creatine Kinase Isoenzyme; UGLU, urine glucose; P-R, PR interval; QTc, QTc interval; BBB, bundle-branch-block; LAFB, left anterior branch block; LPFB, left posterior branch block; LBBB, left bundle branch block; RBBB, right bundle branch block; LVEF, left ventricular ejection fraction; FS, fraction shortening; E/A, mitral valve peak velocity early diastolic filling (E wave) to peak velocity of late diastolic filling (A wave) ratio; Dt, E deceleration time; LVEDD, left ventricular end-diastolic diameter; IVST, interventricular septum thickness; LVPWT, left ventricular posterior wall thickness; LA, left atrium diameter; RA (up and down), right atrium up and down diameter; RA (right and left), right atrium right and left diameter; PA, pulmonary artery internal dimension; Vpa, Pulmonary peak flow rate; Vao, Peak aortic velocity; LAD, left anterior descending; LCX, left circumflex artery; RCA, right coronary artery; LM, left main coronary artery

### Machine learning

#### Feature selection

Feature selection is done after fine-tuning the hyperparameters defined as model parameters, which are assigned arbitrary values before the start of the learning process. During training, Random Forest generates several random decision trees, which are applied to a subset of the data. Random forest checks all the binary results of these decision trees and selects their results by majority voting. Based on the ranking of features with reduced Gini impurity, the degree of reduction in Gini impurity predicted when specific features are removed is calculated. This Gini impurity is then compared with the Gini impurity obtained by using all the characteristics, and this difference is regarded as the importance of the specific characteristic: the more the Gini impurity decreases, the more important the characteristic is. The specific parameters can be seen in Table 2. From this, we get the importance ranking of features. In addition, to make the ML model interpretable, we use the SHAP method to show the importance of features. In the end, we selected the top 15 variables, and the cutoff point was selected based on optimizing the predictive performance of the model with the fewest variables (feature importance ranking see Additional file 2).

**Table 2:** RF parameters

Criterion	Gini
Random state	35
Max depth	3
Min samples leaf	6
Max features	40
N estimators	22

#### Model construction

**Fig. 1:**
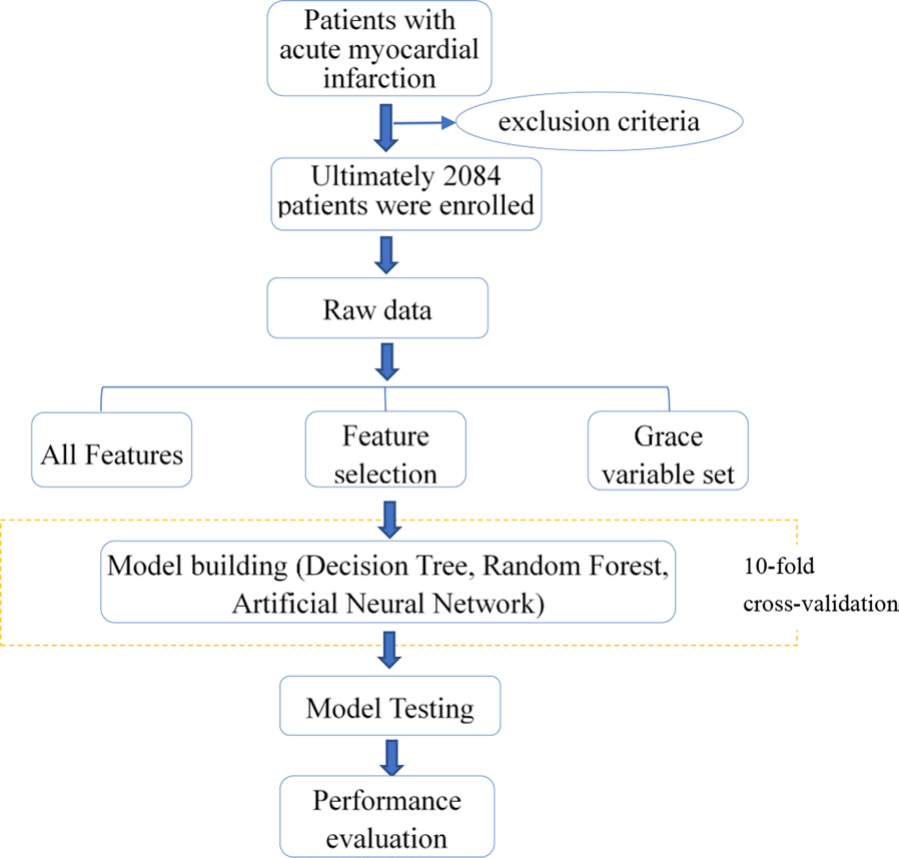
Flow diagram showing the process for evaluating the performance of ML methods

Predictive classifiers were developed based on data from the training set using 3 supervised ML methods: (1) Decision Tree, (2) RF, (3) ANN. We chose 80% as the training set and 20% as the testing set. We use the tenfold cross-validation technique on the training set. The dataset is randomly divided into 10 equal folds, each with approximately the same number of events; 10 validation experiments are then performed, with each fold used in turn as the validation set, and the remaining 9 folds as the training set. Then use the 20% testing set to evaluate model performance (Fig. 1, Additional file 3 describes the detailed data).

**Fig. 2:**
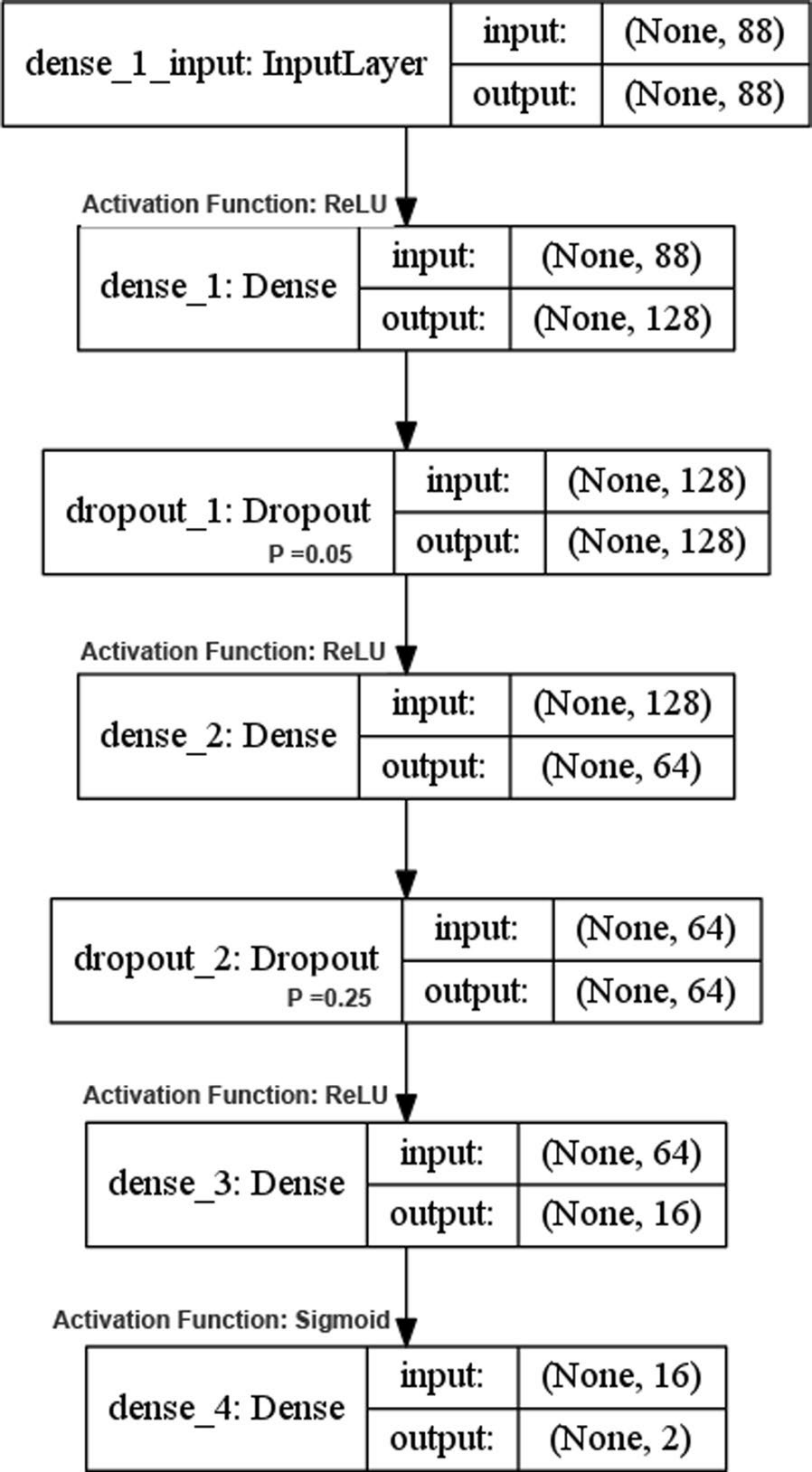
Artificial neural network architecture diagram

The artificial neural network architecture diagram is shown in Fig. 2. The first dense layer uses ReLU as the activation function, and the probability of dropout is 0.05; the second dense layer uses ReLU as the activation function, and the probability is 0.25; the third dense layer uses ReLU as the activation function, and the fourth dense layer uses Sigmoid As an activation function. The loss function is cross-entropy, and the optimization algorithm is RMSProp.

First, we feed all the variables into machine learning to build the prediction model. However, considering that it is difficult for doctors to consider all 45 variables in the actual clinical environment. To simplify the ML model for clinical use, a simplified model is derived from the complete model, which includes the top 15 variables selected based on the RF. Finally, to evaluate the ML model's clinical significance, we input the GRACE risk score variables into three ML algorithms for training to build the GRACE variable set model. The overall performance of the prediction model on the test set was assessed by calculation of accuracy, specificity, false-negative rate, false-positive rate, and the area under the curve (AUC) and the associated 95% CI. We drew receiver operating characteristic (ROC) curves of all models and used the Yoden index to get the best threshold of ROC curves. The ML techniques were implemented in the open-source Python 3.7 environment.

### Statistical analysis

Descriptive analyses and comparisons between clinically defined groups were performed using SPSS 25.0 (IBM, Inc, Chicago, IL, USA). Continuous variables are presented as mean ± SD or median (25th and 75th percentiles) and categorical variables as number and percentage. Baseline characteristics of groups were compared using unpaired t-test or Mann–Whitney’s U-test for continuous variables and by chi-square test for categorical variables. Logistic regression was used to determine the risk of important features of arrhythmia after AMI.A probability value of less than 0.05 was considered statistically significant.

## Results

### Patient characteristics

Excluding patients with incomplete data records and prior arrhythmias, the study included 2084 patients with AMI, of whom 1224 had no arrhythmias and 860 had tachyarrhythmia (611 men and 249 women). Tables 3 and 4 summarizes the differences in demographics, baseline characteristics of admission, laboratory characteristics, echocardiographic parameters, and angiography features between the two groups. (* means* P* < 0.05, ** means *P* < 0.01). Details on all 45 features are available in Additional file 4.

**Table 3:** Comparison of basic characteristics between the two groups

Characteristics	Arrhythmia		
	No (*n* = 1224)	Yes (*n* = 860)	*P* value
*Demographics and history*			
Age, years	57.97 ± 11.38	61.68 ± 10.81	< 0.001**
Sex (male), n (%)	942 (76.96)	611 (71.05)	0.002**
Prior MI, n (%)	86 (7.03)	81 (9.42)	0.048*
*At admission*			
Heart rate, beats/min	75 (66, 86)	72 (62, 85)	< 0.001**
SBP, mmHg	130 (117 ~ 150)	127 (112 ~ 144)	< 0.001**
DBP, mmHg	83.84 ± 15.86	79.59 ± 16.68	< 0.001**
*Laboratory values*			
Pro-BNP (pg/mL)	1003.5 (420.0, 2207.0)	1202.0 (482.0, 2641.5)	0.003**
TNI (μg/L)	19.810 (1.1, 50.0)	30.785 (2.0, 54.0)	0.001**
CK-MB (μg/L)	74.0 (25.4, 161.6)	91.5 (33.7, 190.6)	0.001**
TG (mmol/L)	1.95 ± 1.15	1.82 ± 1.10	0.014*
Cr (μmol/L)	75.88 ± 37.08	79.88 ± 42.88	0.027*
*Findings on ECG*			
BBB, n (%)			< 0.001**
LAFB, n (%)	214 (17.48)	175 (20.35)	
LBBB, n (%)	22 (1.80)	19 (2.21)	
LPFB, n (%)	49 (4.00)	35 (4.07)	
RBBB, n (%)	25 (2.04)	51 (5.93)	
LAFB + RBBB, n (%)	13 (1.06)	27 (3.14)	
Q-Tc (ms)	439 (418, 462)	442 (418, 469)	0.014*

* means* P* < 0.05, ** means *P* < 0.01

**Table 4:** Comparison of the results of echocardiography and PCI between the two groups

Characteristics	Arrhythmia		
	No (*n* = 1224)	Yes (*n* = 860)	*P* value
*Echocardiographic*			
LVEF (%)	52.28 ± 9.04	51.17 ± 9.20	0.006**
RA (up and down), mm	44.0 (42.0, 46.0)	45.0 (42.0, 46.0)	0.006**
RA (right and left), mm	34.0 (32.0, 35.0)	34.0 (32.0, 36.0)	0.003**
Ventricular wall motion abnormal, n (%)			< 0.001**
≥ 2 walls	270 (22.06)	213 (24.77)	
Anterior	430 (35.13)	217 (25.23)	
Apex	19 (1.55)	3 (0.35)	
Anteroseptal	19 (1.55)	2 (0.23)	
Posterior	148 (12.09)	135 (15.70)	
Inferior	297 (24.26)	268 (31.16)	
*Angiographic*			
Lesions vessels, n (%)			< 0.001**
LAD	262 (21.41)	95 (11.05)	
LCX	46 (3.76)	20 (2.33)	
RCA	78 (6.37)	112 (13.02)	
LM	38 (3.10)	35 (4.07)	
LAD + LCX	158 (12.91)	64 (7.44)	
LAD + RCA	198 (16.18)	166 (19.30)	
RCA + LCX	66 (5.39)	60 (6.98)	
Triple vessels	378 (30.88)	308 (35.81)	

* means* P* < 0.05, ** means *P* < 0.01

### ML analysis

#### Variable selection

**Fig. 3:**
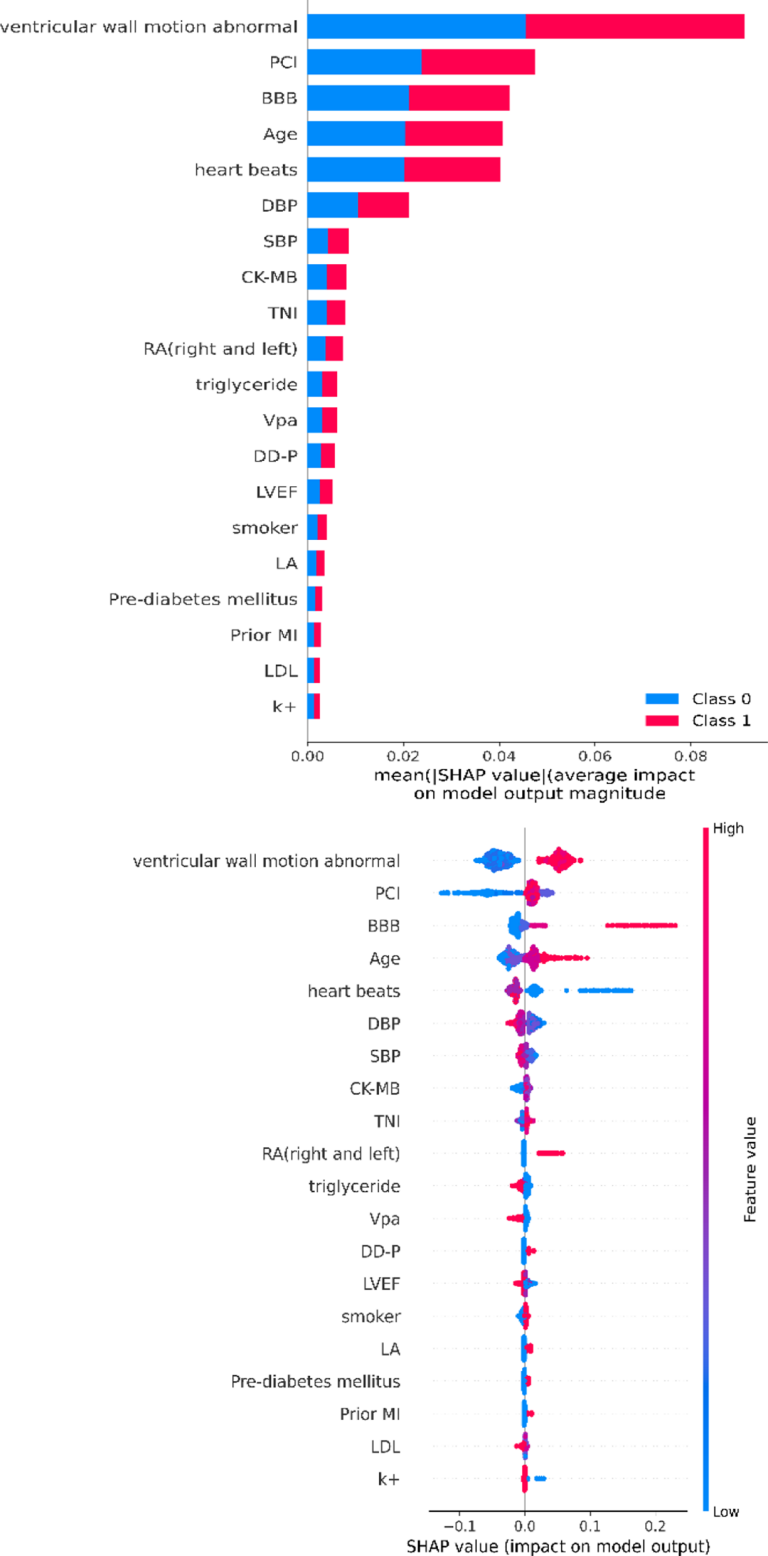
Feature importance

ML extracted top-15 feature-ranking with the random forest for further modeling. After applying SHAP to make the model interpretable, the most important features are abnormal wall motion, lesion location, bundle branch block, age, and heart rate (Fig. 3).

#### Model evaluation and comparison

We use three ML algorithms to build a predictive model of tachyarrhythmia after AMI. Whether it is all variables, 15 important variables, or the GRACE variable set, ANN has better performance than the other two algorithms. The model constructed by the feature selection combined with the ANN algorithm has the best performance, with an accuracy rate of 0.668 (95% CI, 0.621–0.714), which is higher than the Grace variable set model, with an accuracy of 0.644 (95% CI, 0.615–0.673). Table 5 summarizes the accuracy, specificity, false-negative rate, false-positive rate, and the area under the curve (AUC) and the associated 95% CI of each model.

**Table 5:** Predictive performance of all machine learning models

Models	Accuracy	AUC	Specificity	False negative rate	False positive rate
*All features*					
Decision tree	0.627 (95% CI, 0.598–0.656)	0.575 (95% CI, 0.545–0.603)	0.963	0.915	0.037
Random forest	0.646 (95% CI, 0.617–0.675)	0.596 (95% CI, 0.567–0.652)	0.869	0.755	0.131
Artificial neural network	0.650 (95% CI, 0.607–0.675)	0.625 (95% CI, 0.579–0.672)	0.861	0.665	0.139
*Feature selection*					
Decision tree	0.642 (95% CI, 0.613–0.671)	0.592 (95% CI, 0.563–0.648)	0.963	0.915	0.037
Random forest	0.648 (95% CI, 0.601–0.695)	0.605 (95% CI, 0.558 –0.652	0.913	0.802	0.087
Artificial neural network	**0.668 (95% CI, 0.621–0.714)**	**0.654 (95% CI, 0.625–0.683)**	0.922	0.755	0.078
*Grace variable sets*					
Decision tree	0.622 (95% CI, 0.576–0.668)	0.554 (95% CI, 0.508–0.601)	0.973	0.927	0.027
Random forest	0.627 (95% CI, 0.598–0.656)	0.575 (95% CI, 0.545–0.603)	0.966	0.904	0.034
Artificial neural network	0.644 (95% CI, 0.615–0.673)	0.594 (95% CI, 0.565–0.65)	0.892	0.778	0.108

**Fig. 4:**
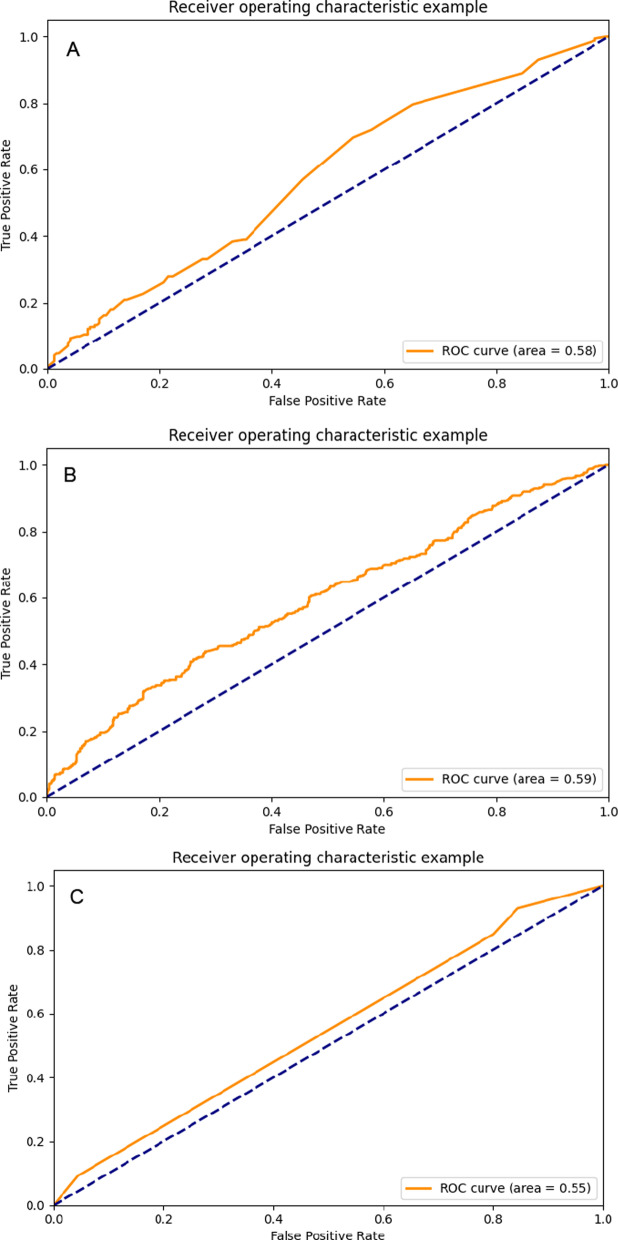
The ROC curves of decision tree models: **A** decision tree-all feature model; **B** decision tree-feature selection model; **C** decision tree-GRACE model;

**Fig. 5:**
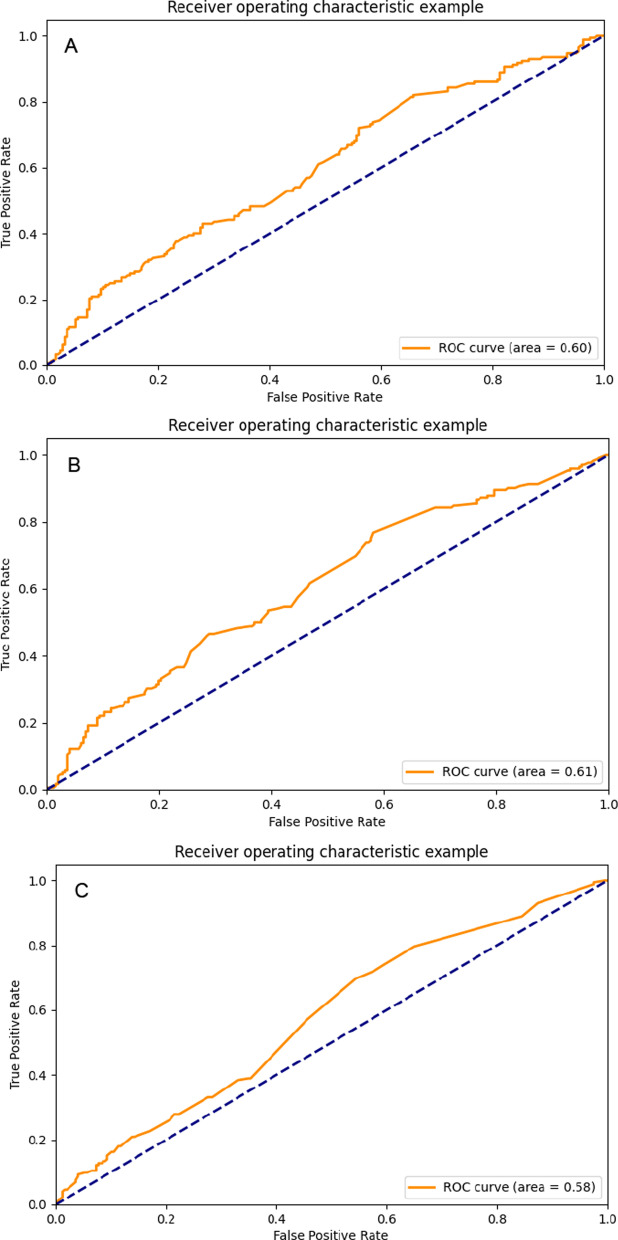
The ROC curves of random forest models: **A** random forest-all feature models; **B** random forest -feature selection model; **C** random forest-GRACE model;

**Fig. 6:**
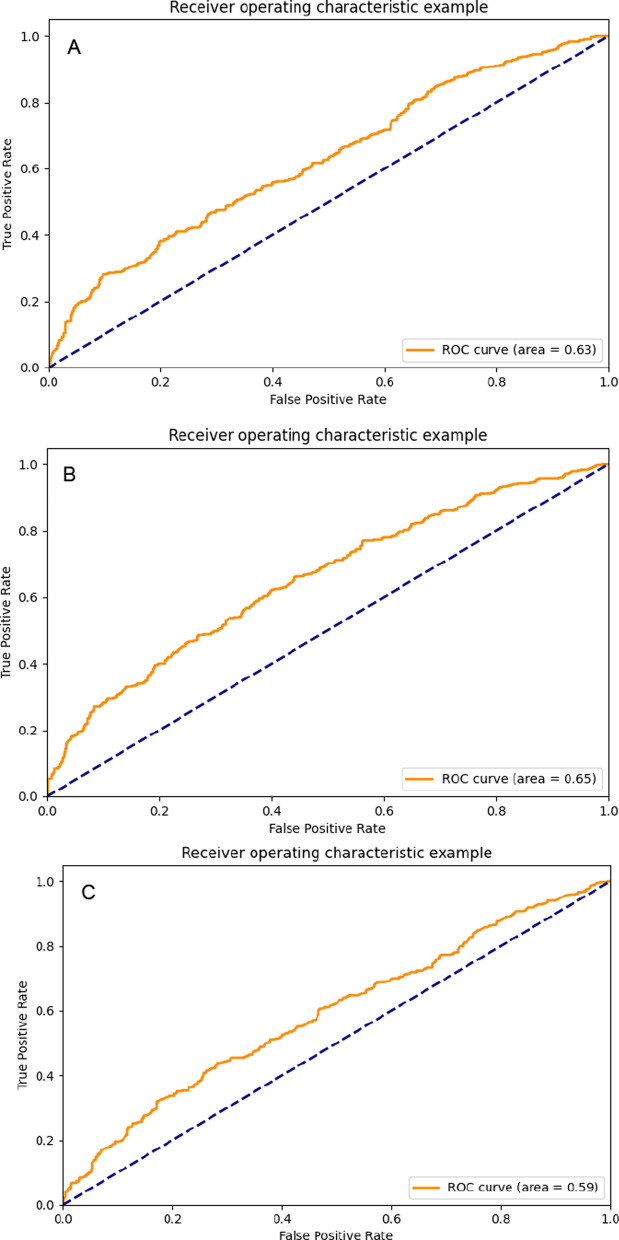
The ROC curves of ANN models: **A** ANN-all feature model; **B** ANN-feature selection model; **C** ANN-GRACE model

We drew ROC curves of all models. Figure 4 is the ROC curve obtained by the decision tree learning three types of data sets. Figure 5 is the ROC curve obtained by RF learning three types of data sets; Fig. 6 is the ROC curve obtained by ANN learning three types of data sets. We can see that the highest value of the area under the ROC curve of the model constructed by the artificial neural network combined with the feature selection variable set is 0.654 (95% CI, 0.625–0.683).

To further explore the clinical application value of ML, we used logistic regression to analyze the risk of important features of arrhythmia after AMI. The results showed RBBB (OR: 4.21; 95% CI: 2.42–7.02), ≥ 2 ventricular wall motion abnormalities (OR: 3.26; 95% CI: 2.01–4.36), and right coronary artery occlusion (OR: 3.00; 95% CI: 1.98–4.56) are important factors related to arrhythmia after AMI (Table 6).

**Table 6:** Odds ratio for important characteristics

Characteristics	OR (95% CI)	*P* value
Age (+ 10-year increments)	1.25 (1.15–1.36)	< 0.001**
Heart rate (+ 20-beat increments)	1.12 (1.06–1.24)	0.047*
*BBB*		
None	1.00 (Dummy variable)	
LAFB	1.33 (1.05–1.68)	0.018*
LBBB	1.22 (0.63–2.34)	0.554
LPFB	1.48 (0.93–2.36)	0.101
RBBB	4.21 (2.42–7.02)	< 0.001**
LAFB + RBBB	4.36 (2.17–8.74)	< 0.001**
*Ventricular wall motion abnormal*		
≥ 2 walls	3.26 (2.01–4.36)	< 0.001**
Anterior		
	1.24 (0.71–2.19)	0.453
Apex	0.32 (0.08–1.23)	0.980
Anteroseptal	0.20 (0.04–1.02)	0.054
Posterior	1.41 (0.78–2.54)	0.254
Inferior	1.21 (1.12–2.24)	0.013*
None	1.00 (Dummy variable)	
*Lesions vessels*		
LAD	1.00 (Dummy variable)	
LCX	0.63 (0.33–1.19)	0.154
RCA	3.00 (1.98–4.56)	< 0.001**
LM	1.77 (1.03–3.06)	0.040*
LAD + LCX	0.91 (0.62–1.35)	0.650
LAD + RCA	1.86 (1.33–2.60)	< 0.001**
RCA + LCX	1.91 (1.20–3.03)	0.006*
Triple vessels	1.67 (1.22–2.64)	0.001*

* means* P* < 0.05, ** means* P* < 0.01

## Discussion

AMI is a clinically critical illness, and the mortality rate after PCI can still reach 10% [3]. Arrhythmia after AMI complicates the patient's condition and increases the Incidence of adverse events (including stroke [31], higher use of pacemakers [4], re-infarction, cardiogenic shock, heart failure, asystole [8], and sudden cardiac death [32]). The hospital mortality of patients with arrhythmia [4, 6, 31, 33], 30-day mortality [34, 35], and 1-year mortality [8] are significantly higher than patients without arrhythmia. In addition, studies have found that in patients undergoing PCI treatment, arrhythmias occurring before and after cardiac catheterization are associated with increased mortality [7]. Therefore, it is essential to predict the occurrence of arrhythmia after AMI as early as possible. To this end, a large number of studies have analyzed the risk factors for arrhythmia after AMI [7, 8, 10, 11, 34, 36–41], but there is no systematic risk model. Currently, AMI's clinical risk model is mainly the GRACE risk score recommended by the ACC/AHA guidelines [42]. Still, it is mainly used to assess patients' mortality and may not accurately predict the occurrence of arrhythmia. Besides, the model is constructed using traditional statistical methods and only linearly analyzes the relationship between a few factors, does not explore the potential prognostic value of interactions between several unexpected weaker risk factors and the primary outcome. For complex diseases, multi-factor and multi-level interactions need to be analyzed. In this case, ML can provide a useful alternative when encountering a large number of potentially relevant variables when building a predictive model. In the cardiovascular field, ML has been used in medical image analysis [43–49], disease classification and diagnosis [16, 19, 50, 51], and predictive model construction [21, 25, 28, 52, 53]. At present, researches related to ML and AMI were mainly devoted to the prediction of patient mortality [25, 54], and the ML model of arrhythmia after AMI has not been explored. In this study, we collected big clinical data of 2084 AMI patients and applied the power of ML to develop predictive models of tachyarrhythmia after AMI.

Before ML, we included 45 variables based on the current AMI risk score [1, 35, 55–59] and the risk factors for tachyarrhythmia after AMI identified in previous studies [7–9, 11, 35–38, 60, 61]. First, we applied 3 ML techniques (decision tree, RF, ANN) combined with all 45 variables to assess the risk of tachyarrhythmia after AMI. Our goal is to accurately predict the patient's arrhythmia with as few features as possible, so we further used the top 15 highly predictive variables to build the ML model. We found that compared with other machine classifiers, the ANN algorithm has better predictive ability in the full-variable model, the important variable model, and the Grace variable model. Surprisingly, after feature selection, the ANN model obtained the best prediction performance. Finally, to evaluate the clinical efficacy of ML, we introduced the widely used GRACE risk variable set (including age, heart rate, blood pressure, Killip grade, ECG changes, myocardial enzymes, serum creatinine, and past medical history) to construct the model. The best accuracy obtained is lower than the feature selection-ANN model. It can be seen that the feature selection-ANN model has higher performance in predicting the occurrence of arrhythmia in the acute phase of AMI.

In terms of variable selection, we combine advanced ML algorithms to perform complex nonlinear analysis on important variables with significant predictive capabilities. In addition, to make the ML model interpretable, we use the SHAP method to show the importance of features. The top five are abnormal wall motion, lesion location, bundle branch block, age, and heart rate. Consistent with the results of previous studies, age, heart rate [8], inferior MI, RCA lesions [9], RBBB, and RBBB + LAFB [62] are related to the occurrence of an arrhythmia, proving that ML has a very reliable Clinical practice. More importantly, the lesion location, abnormal wall motion, and bundle branch block not included in the GRACE score rank the top three in ML, which means that the ML model we constructed is more suitable for predicting arrhythmia in the acute phase of AMI. Abnormal wall motion, bundle branch block, age, and heart rate are easily obtained clinically and can be used as key indicators for CCU physicians to monitor AMI patients. As mentioned above, the occurrence of arrhythmia after PCI can also increase the mortality of patients. Even after revascularization, stricter observations should be made based on the location of the lesion after PCI.

Our results show that the overall performance of ML was moderate, and therefore, it probably cannot yet replace diagnostic or risk estimations that further workup can provide. Nevertheless, when results were compared to those of utilizing the sets of variables considered in the Grace models, ML exhibited a higher performance for predicting the occurrence of tachyarrhythmia after AMI. Therefore, the ML model is more suitable for predicting arrhythmia after AMI than the Grace model and can be used to refine and supplement the current AMI risk score to help clinicians perform a more accurate risk assessment and timely treatment.

## Limitation

The present study naturally carries the limitations of any observational study. However, this kind of largescale retrospective analysis is the main target of the data-driven approaches of ML. Second, this ML approach still needs further model training, validation, and optimization before clinical application. Patients in this study were enrolled from a single center that included only Chinese patients. Nevertheless, we compared the performance of advanced ML algorithms with the GRACE variable set model. The main finding of the current analysis was that ANN exhibited the highest prediction performance. ML-based prediction model could represent a great supplement in optimizing risk assessment and even clinical alerts of patients after AMI.

## Conclusions

In summary, we used advanced ML algorithms to select 15 clinical variables and constructed a prediction model for the occurrence of tachyarrhythmias after AMI. This novel approach proved is superior to the method of the GRACE model. Early prediction of the occurrence of tachyarrhythmias in the acute phase of AMI is critical to clinicians' decision-making. This study highlights the utility of using ML methods for more precise risk assessment.

## Perspectives

We established ML-based prediction models in a cohort of patients with AMI. The GRACE variable set model's comparable performance indicates ML approaches' potential value for evaluating complex and multifactorial diseases. There is no doubt that 2020 has been a great year, dominated by the COVID-19 pandemic. Under these difficult circumstances, most areas of cardiovascular research compromised due to national lockdowns. ML to extract and analyze large volumes of data remotely allowed cardiovascular medicine to continue its evolution. This study is only a small part of this booming field, providing new ideas for what will come to clinical practice in the coming years.

## Supplementary Information

**Additional file 1.** Convert continuous variables to ordinal categorical variables.

**Additional file 2.** Feature importance ranking.

**Additional file 3.** ANN 10-fold cross-validation model.

**Additional file 4.** Differences in all 45 characteristics between the two groups.

## Data Availability

All data generated or analyzed during this study are included in this published article [and its supplementary information files].

## Abbreviations

ACC: American College of Cardiology
AHA: American Heart Association
AMI: Acute Myocardial Infarction
ANN: Artificial Neural Network
AUC: Area Under The Curve
BNP: Brain Natriuretic Peptide
GRACE: Global Registry of Acute Coronary Events
ML: Machine Learning
TNI: Troponin I
TNT: Troponin T
