# Supplementary material for: Application of machine learning to predict the occurrence of arrhythmia after acute myocardial infarction
Source: BMC Med Inform Decis Mak. 2021 Nov 2;21:301. doi: 10.1186/s12911-021-01667-8 (PMC8560220; doi:10.1186/s12911-021-01667-8)
Supplement: Supplementary file 2 — Additional file 2. Feature importance ranking. [file 12911_2021_1667_MOESM2_ESM.pdf]

First, we give the RF parameters in the table below.

Then three types of graphs related to RF features are listed: 1) feature importance ranking graph; 2) taking the average of the absolute value of each feature's SHAP as the feature importance, and obtaining a histogram; 3) reflecting each feature A honeycomb diagram of the importance of features.

RF parameters	
Criterion	Gini
Random state	35
Max depth	3
Min samples leaf	6
Max features	40
N estimators	22

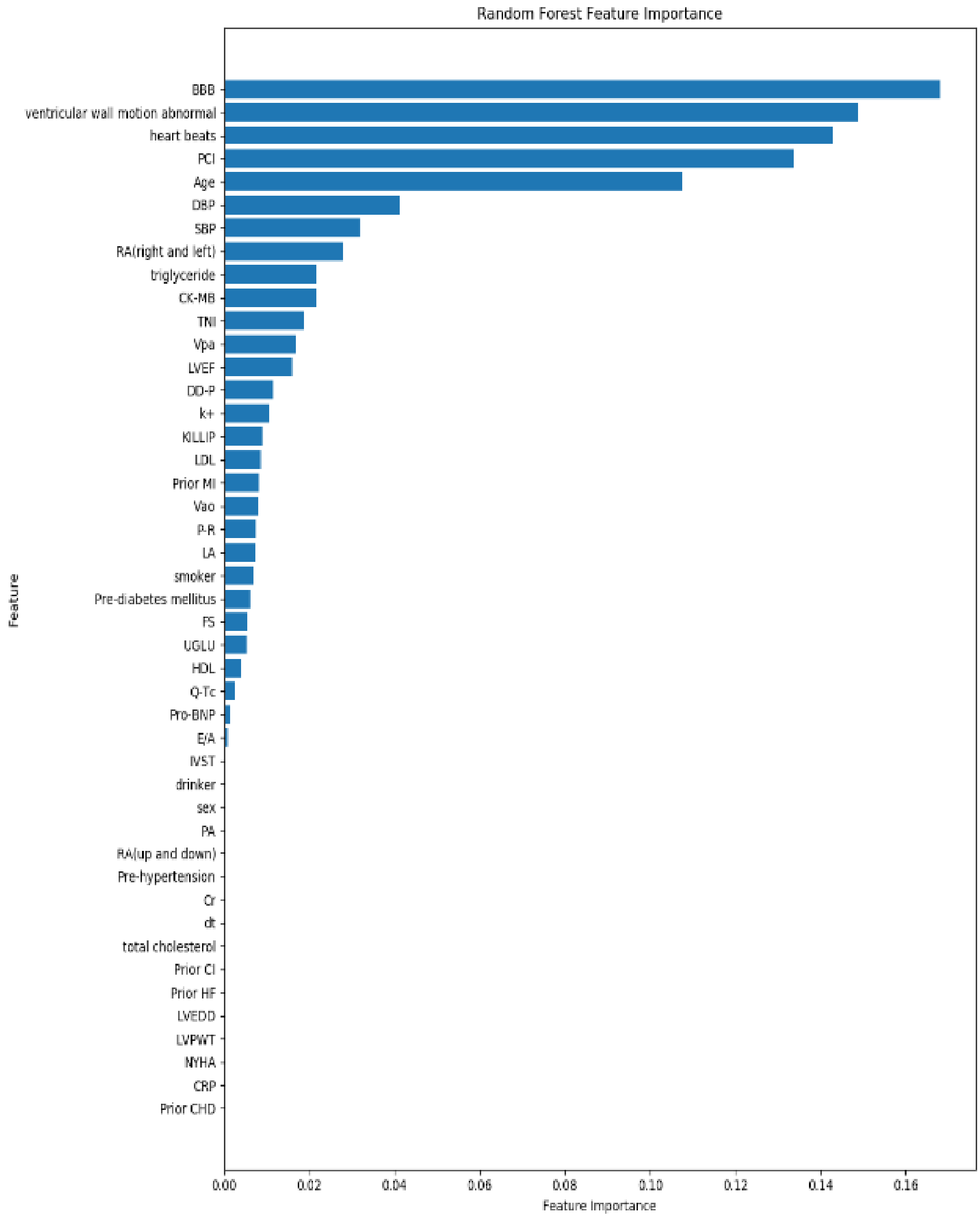

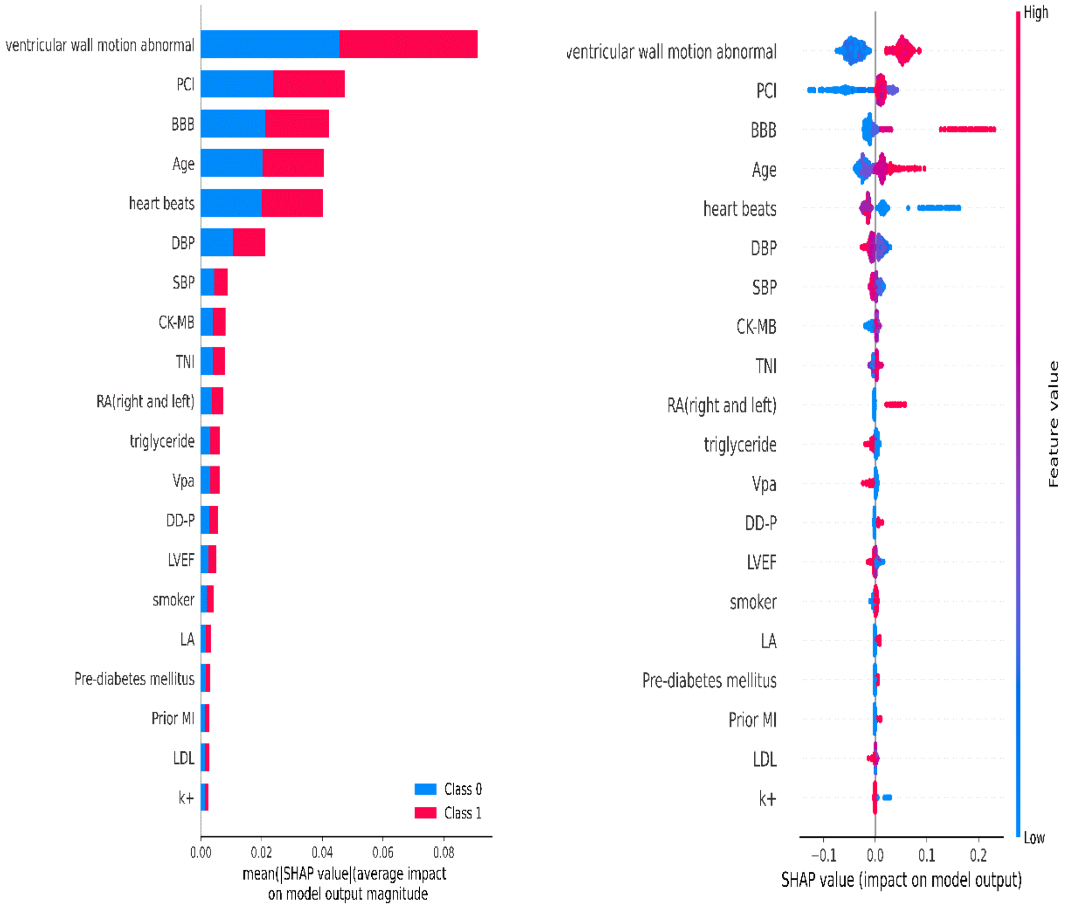
ventricular wall motion abnormal
PCI
BBB
Age
heart beats
DBP
SBP
CK-MB
TNI
RA(right and left)
triglyceride
Vpa
DD-P
LVEF
smoker
LA
Pre-diabetes mellitus
Prior MI
LDL
k+
Class 0
Class 1
0.00
0.02
0.04
0.06
0.08
mean(|SHAP value|(average impact
on model output magnitude
ventricular wall motion abnormal
PCI
BBB
Age
heart beats
DBP
SBP
CK-MB
TNI
RA(right and left)
triglyceride
Vpa
DD-P
LVEF
smoker
LA
Pre-diabetes mellitus
Prior MI
LDL
k+
−0.1
0.0
0.1
0.2
SHAP value (impact on model output)
High
Low
Feature value
