# Supplementary material for: Application of machine learning to predict the occurrence of arrhythmia after acute myocardial infarction
Source: BMC Med Inform Decis Mak. 2021 Nov 2;21:301. doi: 10.1186/s12911-021-01667-8 (PMC8560220; doi:10.1186/s12911-021-01667-8)
Supplement: Supplementary file 3 — Additional file 3. ANN 10-fold cross-validation model. [file 12911_2021_1667_MOESM3_ESM.pdf]

ANN 10-fold cross-validation model

All features：

The results were cross-verified ten times

{'acc': 0.667664647102356, 'auc': 0.6609997093868061, 'tnr': 0.8602150537634409, 'fnr': 0.581081081081081, 'frr': 0.13978494623655913, 'loss': 0.6819909436260155},

{'acc': 0.682634711265564, 'auc': 0.652002427184466, 'tnr': 0.8349514563106796, 'fnr': 0.5625, 'frr': 0.1650485436893204, 'loss': 0.6951385143988147},

{'acc': 0.6467065811157227, 'auc': 0.6288145539906104, 'tnr': 0.8541666666666666, 'fnr': 0.6338028169014085, 'frr': 0.14583333333333334, 'loss': 0.7342561394154669},

{'acc': 0.727544903755188, 'auc': 0.7017345399698341, 'tnr': 0.9019607843137255, 'fnr': 0.5538461538461539, 'frr': 0.09803921568627451, 'loss': 0.6509591858544036},

{'acc': 0.658682644367218, 'auc': 0.6521422325852521, 'tnr': 0.7872340425531915, 'fnr': 0.5068493150684932, 'frr': 0.2127659574468085, 'loss': 0.7267280610021717},

{'acc': 0.6766467094421387, 'auc': 0.6767676767676768, 'tnr': 0.9292929292929293, 'fnr': 0.6764705882352942, 'frr': 0.0707070707070707, 'loss': 0.6641568363069774},

{'acc': 0.697604775428772, 'auc': 0.5816199376947041, 'tnr': 0.9532710280373832, 'fnr': 0.7666666666666667, 'frr': 0.04672897196261682, 'loss': 0.8866584267088039},

{'acc': 0.7065868377685547, 'auc': 0.654179104477612, 'tnr': 0.83, 'fnr': 0.47761194029850745, 'frr': 0.17, 'loss': 0.7221663408650609},

{'acc': 0.7048192620277405, 'auc': 0.6977987421383647, 'tnr': 0.9150943396226415, 'fnr': 0.6666666666666666, 'frr': 0.08490566037735849, 'loss': 0.6783635968185333},

{'acc': 0.6445783376693726, 'auc': 0.6621212121212121, 'tnr': 0.83, 'fnr': 0.6363636363636364, 'frr': 0.17, 'loss': 0.6668690198875336}]

The average results

{'avg_acc': 0.6813469409942627, 'avg_auc': 0.6568180136316539, 'avg_tnr': 0.8696186300560658, 'avg_fnr': 0.6061858865127908, 'avg_frr': 0.13038136994393418, 'avg_loss': 0.7107287064883783}

The test results

{'acc': 0.65016746163368225, 'auc': 0.624194229351032449, 'tnr': 0.8545132743362832, 'fnr': 0.6650833333333334, 'frr': 0.13858672566371684, 'loss': 0.7002809304369694}

Feature selection

The results were cross-verified ten times

[{'acc': 0.652694582939148, 'auc': 0.673278116826504, 'tnr': 0.7634408602150538, 'fnr': 0.4864864864864865, 'frr': 0.23655913978494625, 'loss': 0.6744846376830232},
{'acc': 0.6946107745170593, 'auc': 0.7097997572815533, 'tnr': 0.8155339805825242, 'fnr': 0.5, 'frr': 0.18446601941747573, 'loss': 0.6359324869281517},
{'acc': 0.6467065811157227, 'auc': 0.646200117370892, 'tnr': 0.9270833333333334, 'fnr': 0.7323943661971831, 'frr': 0.07291666666666667, 'loss': 0.6889348776040677},
{'acc': 0.697604775428772, 'auc': 0.7018099547511313, 'tnr': 0.9117647058823529, 'fnr': 0.6307692307692307, 'frr': 0.08823529411764706, 'loss': 0.6458202910994342},
{'acc': 0.6946107745170593, 'auc': 0.6967356455843778, 'tnr': 0.8723404255319149, 'fnr': 0.5342465753424658, 'frr': 0.1276595744680851, 'loss': 0.6660355375912375},
{'acc': 0.658682644367218, 'auc': 0.6697118241235889, 'tnr': 0.9393939393939394, 'fnr': 0.75, 'frr': 0.06060606060606061, 'loss': 0.6666583932802349},
{'acc': 0.682634711265564, 'auc': 0.6333333333333334, 'tnr': 0.8691588785046729, 'fnr': 0.65, 'frr': 0.1308411214953271, 'loss': 0.6716121149634173},
{'acc': 0.6706587076187134, 'auc': 0.6497014925373135, 'tnr': 0.87, 'fnr': 0.6268656716417911, 'frr': 0.13, 'loss': 0.675612704482621},
{'acc': 0.7289156913757324, 'auc': 0.7317610062893082, 'tnr': 0.8773584905660378, 'fnr': 0.5333333333333333, 'frr': 0.12264150943396226, 'loss': 0.6136133038854025},
{'acc': 0.6686747074127197, 'auc': 0.6887121212121212, 'tnr': 0.82, 'fnr': 0.5606060606060606, 'frr': 0.18, 'loss': 0.6470056556793581}]

The average results

{'avg_acc': 0.6895793950557709, 'avg_auc': 0.6801043369310124, 'avg_tnr': 0.866607461400983, 'avg_fnr': 0.6004701724376551, 'avg_frr': 0.1333925385990171, 'avg_loss': 0.6585710003196947}

The test results

{'acc': 0.66833014154434204, 'auc': 0.6544010877581121, 'tnr': 0.92254867256637168, 'fnr': 0.755375, 'frr': 0.07825132743362832, 'loss': 0.691482071956379}

Grace Variable set

The results were cross-verified ten times

[{'acc': 0.6407185792922974, 'auc': 0.6648503342051729, 'tnr': 0.8279569892473119, 'fnr': 0.581081081081081, 'frr': 0.17204301075268819, 'loss': 0.6665298267752825},
{'acc': 0.589820384979248, 'auc': 0.5798695388349515, 'tnr': 0.8155339805825242, 'fnr': 0.765625, 'frr': 0.18446601941747573, 'loss': 0.6709241024748294},
{'acc': 0.56886225938797, 'auc': 0.574163732394366, 'tnr': 0.78125, 'fnr': 0.7183098591549296, 'frr': 0.21875, 'loss': 0.683570411033973},
{'acc': 0.658682644367218, 'auc': 0.6790346907993967, 'tnr': 0.8431372549019608, 'fnr': 0.6307692307692307, 'frr': 0.1568627450980392, 'loss': 0.6521756181459941},
{'acc': 0.589820384979248, 'auc': 0.6348003497522587, 'tnr': 0.8297872340425532, 'fnr': 0.7123287671232876, 'frr': 0.1702127659574468, 'loss': 0.673468379203431},
{'acc': 0.57485032081604, 'auc': 0.5973707664884136, 'tnr': 0.7878787878787878, 'fnr': 0.7205882352941176, 'frr': 0.21212121212121213, 'loss': 0.6746285489933219},
{'acc': 0.6377245783805847, 'auc': 0.5901090342679128, 'tnr': 0.8411214953271028, 'fnr': 0.7166666666666667, 'frr': 0.1588785046728972, 'loss': 0.6657841908717583},
{'acc': 0.6317365169525146, 'auc': 0.6548507462686567, 'tnr': 0.83, 'fnr': 0.6716417910447762, 'frr': 0.17, 'loss': 0.658357067379409},
{'acc': 0.6536144614219666, 'auc': 0.6522012578616352, 'tnr': 0.7830188679245284, 'fnr': 0.5833333333333334, 'frr': 0.2169811320754717, 'loss': 0.651420051793018},
{'acc': 0.6204819083213806, 'auc': 0.6389393939393939, 'tnr': 0.79, 'fnr': 0.6363636363636364, 'frr': 0.21, 'loss': 0.6625863241862102}]

The average results

{'avg_acc': 0.666312038898468, 'avg_auc': 0.6266189844812158, 'avg_tnr': 0.8129684609904769, 'avg_fnr': 0.673670760083106, 'avg_frr': 0.18703153900952307, 'avg_loss': 0.6659444520857227}

The test results

{'acc': 0.6439473652839661, 'auc': 0.5941556231563422, 'tnr': 0.8921849557522124, 'fnr': 0.77822916666666666, 'frr': 0.10811150442477876, 'loss': 0.6937975290289335}
